# L-theanine alleviates myocardial ischemia/reperfusion injury by suppressing oxidative stress and apoptosis through activation of the JAK2/STAT3 pathway in mice

**DOI:** 10.1186/s10020-024-00865-0

**Published:** 2024-06-28

**Authors:** Qi Li, Jiaqi Ding, Boyu Xia, Kun Liu, Koulong Zheng, Jingjing Wu, Chao Huang, Xiaomei Yuan, Qingsheng You

**Affiliations:** 1 Department of Cardiothoracic Surgery, Affiliated Hospital of Nantong University, Nantong University, #20 Xishi Road, Nantong, 226001 Jiangsu China; 2 Department of Cardiology, The Second Affiliated Hospital of Nantong University, Nantong, Jiangsu China; 3 Department of Cardiology, Suzhou Kowloon Hospital of Shanghai Jiaotong University School of Medicine, Suzhou, Jiangsu China; 4 Department of Pharmacology, School of Pharmacy, Nantong University, Nantong, Jiangsu China; 5 Department of Cardiology, Sichuan Provincial People’s Hospital, University of Electronic Science and Technology of China, Chengdu, Sichuan China

**Keywords:** L-theanine, Ischemia/reperfusion, Oxidative stress, Inflammation, Apoptosis, JAK2 /STAT3

## Abstract

**Background:**

L-theanine is a unique non-protein amino acid in tea that is widely used as a safe food additive. We investigated the cardioprotective effects and mechanisms of L-theanine in myocardial ischemia-reperfusion injury (MIRI).

**Methods:**

The cardioprotective effects and mechanisms of L-theanine and the role of Janus Kinase 2 (JAK2)/signal transducer and activator of transcription 3 (STAT3) signaling were investigated in MIRI mice using measures of cardiac function, oxidative stress, and apoptosis.

**Results:**

Administration of L-theanine (10 mg/kg, once daily) suppressed the MIRI-induced increase in infarct size and serum creatine kinase and lactate dehydrogenase levels, as well as MIRI-induced cardiac apoptosis, as evidenced by an increase in Bcl-2 expression and a decrease in Bax/caspase-3 expression. Administration of L-theanine also decreased the levels of parameters reflecting oxidative stress, such as dihydroethidium, malondialdehyde, and nitric oxide, and increased the levels of parameters reflecting anti-oxidation, such as total antioxidant capacity (T-AOC), glutathione (GSH), and superoxide dismutase (SOD) in ischemic heart tissue. Further analysis showed that L-theanine administration suppressed the MIRI-induced decrease of phospho-JAK2 and phospho-STAT3 in ischemic heart tissue. Inhibition of JAK2 by AG490 (5 mg/kg, once daily) abolished the cardioprotective effect of L-theanine, suggesting that the JAK2/STAT3 signaling pathway may play an essential role in mediating the anti-I/R effect of L-theanine.

**Conclusions:**

L-theanine administration suppresses cellular apoptosis and oxidative stress in part via the JAK2/STAT3 signaling pathway, thereby attenuating MIRI-induced cardiac injury. L-theanine could be developed as a potential drug to alleviate cardiac damage in MIRI.

**Supplementary Information:**

The online version contains supplementary material available at 10.1186/s10020-024-00865-0.

## Introduction

Ischemic heart disease is a leading cause of death and disability worldwide (Hausenloy and Yellon 2013). In patients with myocardial infarction (MI), the treatment of choice to alleviate the acute ischemic damage to the myocardium and limit the size of the MI is thrombolytic therapy or primary percutaneous coronary intervention (PPCI) (Yellon and Hausenloy 2007). However, restoring blood flow to the ischemic myocardium can lead to myocardial damage or even death. This phenomenon is called myocardial ischemia-reperfusion injury (MIRI), for which there is still no effective therapy (Yellon and Hausenloy 2007; Piper et al. 1998). To reduce the fatal side effects of the reperfusion phase, it is therefore important to introduce an effective cardioprotection strategy.

The pathophysiology of MIRI is multifaceted and includes myocyte apoptosis, necrosis, destructive inflammatory responses, and oxidative stress, which together increase infarct size and cause functional and structural damage to the heart (Sánchez-Hernández et al. 2020). There is growing evidence that the Janus kinase-2/transducer signal transducer and activator of transcription pathway-3 (JAK2/STAT3) signaling pathway is an anti-inflammatory and antioxidant signaling pathway that plays a critical role in the cardioprotective effects of MIRI (Sun and Mao 2018; Schindler 2002). Signal transducer and STAT3 is a signaling molecule and transcription factor that plays an essential protective role in the heart and is activated by phosphorylation of its tyrosine residue 705 (Y705) by receptor and non-receptor tyrosine kinases (Harhous et al. 2019). JAK belongs to a family of four non-receptor tyrosine kinases (JAK1, JAK2, JAK3 and TYK2) that provide docking sites for STATs and selectively phosphorylate them at a single tyrosine residue (Benucci et al. 2022). Activation of JAK2 induces the phosphorylation of STAT3, which in turn binds to specific DNA elements and up-regulates antioxidant and anti-apoptotic genes. This signaling pathway is part of the major survivor activator enhancement pathway (SAFE), whose activity is significantly reduced in I/R injury (Yang 2018; Shanmugam et al. 2021). Protective interventions to activate the JAK2/STAT3 signaling pathway are considered as potential preventive cardioprotective agents for use in cardiac surgery (Schindler et al. 2007; Wang et al. 2016). For example, a recent report confirmed that insulin exerts a cardioprotective effect on the heart through activation of the JAK2/STAT3 signaling pathway, which can be abolished by AG490, a JAK2 inhibitor (Shanmugam et al. 2021). These results suggest the importance of JAK2/STAT3 pathway activation in improving the clinical prognosis of patients with MIRI.

L-theanine, i.e., N-ethyl-G-glutamine, is a unique non-protein amino acid in tea that was first discovered in green tea by Sakato in 1949 (Kawagishi and Sugiyama 1992; Vuong et al. 2011) and has since been widely used as a safe food additive in beverages and foods (Li et al. 2022a, b). It has been widely reported that treatment with L-theanine improves sleep quality (Wei et al. 2022), gastric ulcers (Chatterjee et al. 2014), cerebral ischemia/reperfusion injury (Sun et al. 2013), post-traumatic stress disorder (Ceremuga et al. 2014), etc., suggesting a variety of physiological functions such as anti-inflammatory (Podolsky 2002), anti-apoptotic (Li et al. 2018), antioxidant (Liu et al. 2022), and neuroprotective effects (Takehana et al. 2017). For example, in a dextran sulfate sodium (DSS)-induced colitis model in C57BL/6J mice, treatment with L-theanine had anti-inflammatory effects by decreasing the expression of pro-inflammatory cytokines, proteins and mRNA levels, including interleukin-1β (IL-1β), interleukin-6 (IL-6), and tumor necrosis factor-α (TNF-α), and attenuated DSS-induced colon injury and oxidative stress by increasing GSH content and decreasing MDA content (Wang et al. 2020). Moreover, L-theanine reduced hepatic ischemia-reperfusion injury (HIRI)-induced TNF-α and inducible nitric oxide synthase (iNOS) production and HIRI-induced increase in myeloperoxidase and MDA activities in rats, indicating its protective effect on liver necrosis after HIRI in rats (Küçükaslan et al. 2021). It was also reported that L-theanine attenuated imiquimod-induced psoriasis-like skin inflammation by inhibiting the activation of the NF-κB signaling pathway and lowering the levels of chemokines in mice (Xu et al. 2021). Furthermore, L-theanine can protect H9C2 cells from hydrogen peroxide-induced apoptosis by increasing GSH and SOD levels, thereby enhancing antioxidant capacity (Li et al. 2018).

Recently, L-theanine has been confirmed to regulate the JAK2/STAT3 signaling pathway to attenuate angiotensin II-induced proliferation and migration of vascular smooth muscle cells (Ben et al. 2018). However, to date, the therapeutic effects and potential mechanisms of L-theanine in I/R-induced cardiac injury remain unclear. Therefore, we addressed this question using a model of MIRI in C57BL/6J mice and investigated the effects of L-theanine supplementation on oxidative stress and cellular apoptosis in the ischemic tissue of I/R-stimulated mice.

## Materials and methods

### Animals

Male C57BL6/J mice (6–8 weeks old) were obtained from Vital River Laboratory Animal Technology Co, Ltd (Beijing, China). Mice were kept five per cage for one week under standard laboratory conditions with free access to food and water. All animal experiments were approved by the Animal Ethics Committee of Nantong University (Permit Number: 2,110,836) and were conducted in accordance with the Guidelines for the Use of Animals in Toxicology adopted by the Society of Toxicology in 1999.

### Materials

L-theanine was purchased from Med Chem Express (MCE, New Jersey, USA) and dissolved in a solution of dimethyl sulfoxide (DMSO) with PEG300, Tween-80, and saline (Galvao et al. 2014). The vehicle was a solution containing only DMSO with PEG300, Tween-80 and saline. The final concentration of DMSO is < 0.05%. No toxic effect of DMSO was observed.

### Experimental arrangement and drug treatment

After a 1-week acclimation period, mice were randomly assigned to the Sham group (saline + DMSO), the I/R group, the I/R + L-theanine group, and the I/R + L-theanine + AG490 group (*n* = 5 in each group). L-theanine was administered intraperitoneally at a dose of 10 mg/kg (Sugiyama and Sadzuka 2004; Nagai et al. 2015) and AG490 at a dose of 5 mg/kg (Chen et al. 2020) once daily for seven days, the last administration on the day of surgery.

### Establishment of the myocardial I/R model

Mice were anesthetized with inhalation anesthesia containing 5% isoflurane via an isoflurane delivery system (Yuyan Instruments, Shanghai, China) (Lu et al. 2020). An incision was made in the fourth intercostal space to open the chest. The pleura was punctured to expose the heart. Then, the heart was manually pushed out with pressure on the upper back of the mouse, and a slip knot was tied with a 6 − 0 suture approximately 2 mm from the origin of the left anterior descending artery (LADA) to create ischemia. Effective ligation was confirmed by the appearance of a pale color in the anterior wall of the left ventricle. After the LADA was occluded for 30 min, the ligature was released for 24 h of reperfusion. Mice in the sham groups underwent similar procedures except for closure of the LADA.

### Determination of myocardial infarction size

Evans blue and 2, 3, 5-triphenyltetrazolium chloride (TTC) staining was performed to determine the area of myocardial infarction (Yu et al. 2018). At the end of reperfusion, 1.5 ml of 1% Evans blue was injected into the left ventricle. Then, the heart was rapidly excised, frozen at -20℃, and cut into 4 sections perpendicular to the long axis, resulting in 4 sections per heart. The prepared slides were incubated with 1% TTC at 37℃ for 20 min, and the sections were photographed with a Canon 600D digital single-lens reflex (DSLR) camera.

### Histopathological examination

Histopathological examination of myocardial tissue was performed by hematoxylin-eosin staining (HE) as previously described. Tissue sections were deparaffinized with xylene (5 min × 3 times), followed by incubation in hematoxylin solutions for 5 min. Then the sections were treated with hematoxylin differentiation solution and hematoxylin Scott tap bluing and rinsed with tap water, followed by dehydration in 95% or 85% ethanol (5 min each). Finally, sections were mounted and examined with a light microscope (Nikon Eclipse E100, Nikon, Japan).

### Echocardiographic analysis of cardiac function

After 24 h of reperfusion, cardiac functions of the mice were examined with a small animal ultrasound system (VEVO 2100, VisualSonics, Toronto, Canada). Two-dimensional guided M-mode tracings were recorded in a short-axis view of the left ventricle at the level of the midpapillary muscle, and then the inner diameter of the left ventricle was measured at the end of diastole (LVID-d) and at the end of systole (LVID-s).

### Measurements of malondialdehyde (MDA), glutathione (GSH), nitric oxide (NO), total antioxidant capacity (T-AOC), and superoxide dismutase (SOD)

The concentrations of MDA, GSH, NO, T-AOC, and SOD in tissue homogenates of left ventricles were measured with commercial kits purchased from the Nanjing Jiancheng Bioengineering Institute (Nanjing, China). Results were normalized to total protein using the BCA method (Beyotime, Shanghai, China) and expressed as units NO g protein, µmol MDA, µmol GSH, units SOD, and mmol T-AOC mg protein in the homogenate.

### Measurement of LDH and CK concentrations

Serum lactate dehydrogenase (LDH) and creatine kinase (CK) concentrations were determined using commercial kits purchased from the Nanjing Jiancheng Bioengineering Institute. Results were expressed as U/ml or U/L units.

### Myocardial dihydroethidium (DHE) staining

DHE staining was performed according to the manufacturer’s protocols. Frozen heart Sect. (8 μm thick) were incubated with DHE solution (Servicebio, Wuhan, China) for 30 min at 37 °C, and then DAPI was used to detect the nucleus. Staining images were viewed under a fluorescence microscope (Leica, Wetzlar, Germany).

### Myocardial TUNEL staining

Cellular apoptosis was determined using a One Step TUNEL Apoptosis Assay Kit (Beyotime, Shanghai, China). Cardiac tissues were harvested, fixed with 4% paraformaldehyde (PFA), dehydrated, and embedded in OCT. The frozen tissue was cut into 8 μm-thick slices, treated with PFA, permeabilized with 0.5% Triton X-100, and incubated in a TUNEL reaction buffer at 37 °C for 60 min according to the manufacturer’s instructions. Finally, the glycerol-sealed slides were analyzed using a fluorescence microscope (Leica, Wetzlar, Germany).

### Western blot

Total proteins were extracted from ischemic regions of myocardial tissue. Protein concentrations were determined using BCA kits (Beyotime, Shanghai, China). Equal amounts of protein were separated in a 10% SDS-PAGE gel at 80 V for 1.5 h and transferred to nitrocellulose membranes (Merck Millipore, Darmstadt, Germany) at 260 mA for 90 min. After blocking with nonfat milk at room temperature, the nitrocellulose membranes were incubated overnight at 4 °C with the antibodies against caspase-3 (1:600), BAX (1:500), Bcl-2 (1:500), JAK2 (1:1000), p-JAK2 (1:500), STAT3 (1:1000), p-STAT3 (1:500), and GAPDH (1:8000). On the second day, membranes were washed three times in TBST and treated with IRDye 680-labeled secondary antibody (1:8000) at room temperature for 2 h. Western blot bands were visualized using an Odyssey CLX Western blot detection system (LICOR, Nebraska, USA) and quantified using Image J software.

### Statistical analysis

Statistical analyses were performed using one-way analysis of variance (ANOVA) or two-way ANOVA with repeated measures followed by Bonferroni multiple comparisons via GraphPad Prism 9 (Graphpad Software, CA, USA). Values of *P* < 0.05 were considered statistically significant. Data are expressed as mean ± standard error of the mean (SEM).

## Results

### L-theanine alleviates IR-induced cardiac injury in vivo

**Fig. 1:**
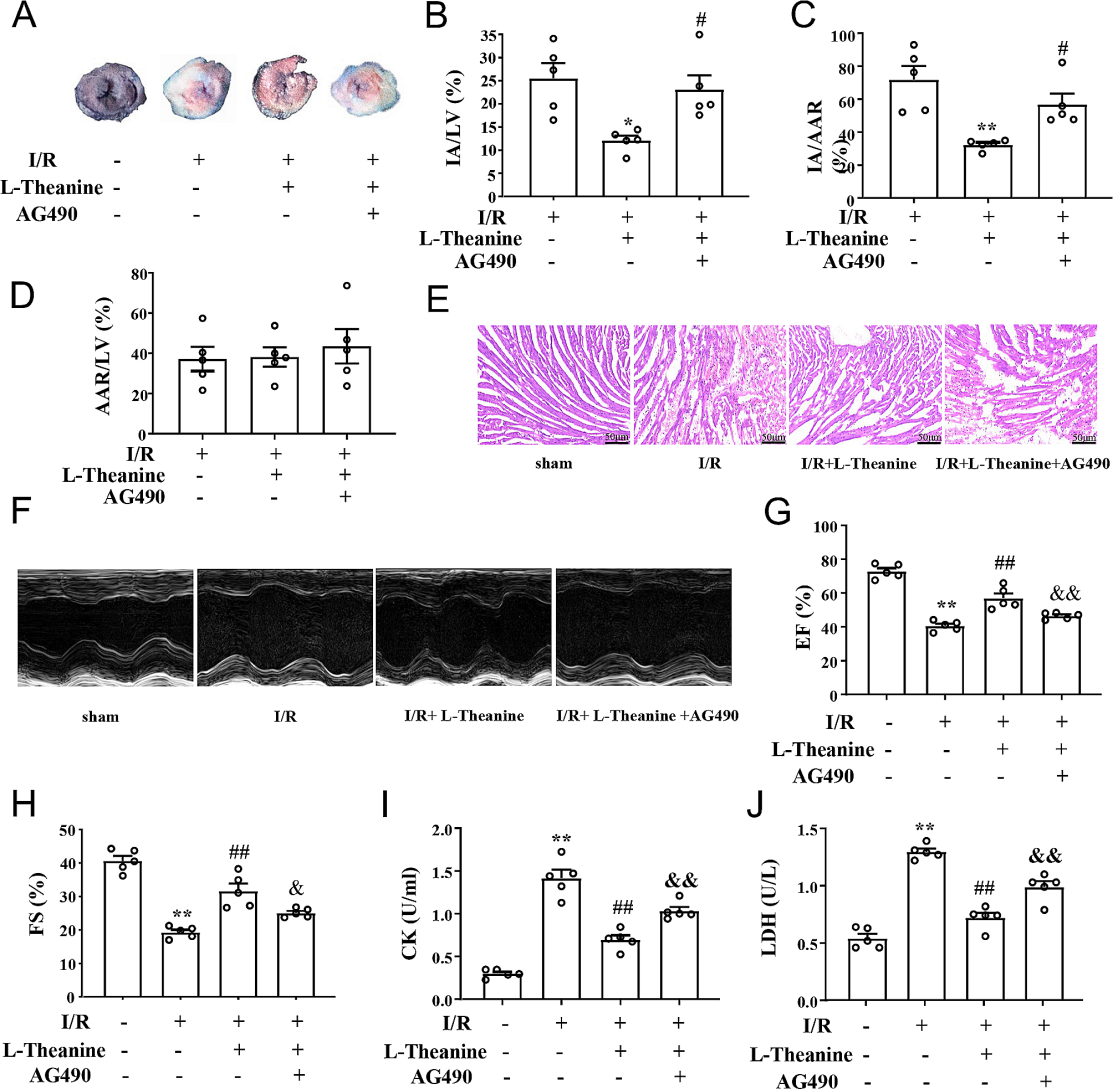
Effect of L-theanine on I/R-induced myocardial damage and cardiac function. **(A)** A representative photograph shows Evans blue/TTC double staining in the heart of mice treated with or without L-theanine (10 mg/kg). The nonischemic area, which was not at risk, was stained blue, whereas the area at risk was red and the infarct area was pale. **(B-D)** Quantitative analysis showed the infarct size of the heart 24 h after reperfusion in mice treated with or without L-theanine: Pretreatment with L-theanine reduced IA/LV in I/R mice from 25.53–12.08% and IA/AAR in I/R mice from 71.81–32.34%, no significant change in AAR/LV in I/R and I/R + L-theanine mice, however, AG490 administration reversed the protective effect of L-theanine (*n* = 5, *p < 0.05 or *p < 0.01 vs. I/R, #*p* < 0.05 vs. I/R + L-theanine); AAR, area at risk; LV, left ventricle; IA, infarct area. **(E)** Representative results of hematoxylin-ecosin (HE) staining results showed that pretreatment with L-theanine reduced I/R-induced cardiac injury in mice, AG490 abolished the effect of L-theanine. Scale bar: 50 μm. **(F)** Representative images show cardiac function determined by echocardiography. **(G, H)** Quantitative analysis showed that L-theanine pretreatment prevented the I/R-induced decrease in ejection fraction (EF, G) and fractional shortening (FS, H) (*n* = 5, **p < 0.01 vs. sham treatment, ##*p* < 0.01 vs. I/R, &*p* < 0.05 or &&*p* < 0.01 vs. I/R + L-theanine). **(I, J)** Quantitative analysis showed that L-theanine pretreatment prevented the I/R-induced increase in serum levels of creatine kinase (CK, I) and lactate dehydrogenase (LDH, J) (*n* = 5, **p < 0.01 vs. sham treatment, ##*p* < 0.01 vs. I/R, &&*p* < 0.01 vs. I/R + L-theanine). Data are expressed as mean ± SEM.

We first investigated the cardioprotective potential of L-theanine in the MIRI model. Pretreatment with L-theanine (10 mg/kg) significantly reduced the I/R-induced infarct area related to the left ventricle from 25.53 to 12.08% (Fig. 1A, B) and the infarct area related to the area at risk from 71.81 to 32.34% (Fig. 1A, C) in I/R-treated mice, whereas application of the JAK2 inhibitor AG490 reversed these effects of L-theanine (Fig. 1B, F_2,12_ = 7.16, *p* < 0.05; Fig. 1C, F_2,12_ = 10.56, *p* < 0.05). H&E staining of cardiac tissue from I/R mice showed increased cross-sectional area of cardiomyocytes compared with sham treatment (Fig. 1E). Pretreatment with L-theanine decreased the cross-sectional area of cardiomyocytes, suggesting amelioration of I/R-induced cardiac tissue injury, but these effects of L-theanine were abolished by JAK2 inhibition with AG490 (Fig. 1E). Furthermore, the cardioprotective effects of L-theanine were examined in I/R mice by echocardiography (Fig. 1F). We found that pretreatment with L-theanine significantly prevented the I/R-induced reduction in left ventricular ejection fraction (EF, Fig. 1G) and fractional shortening (FS, Fig. 1H) in mice, whereas these effects of L-theanine were abolished by AG490 (Fig. 1G, F_3,16_ = 55.88, *p* < 0.01; 1H, F_3,16_ = 41.17, *p* < 0.01). It was also found that pretreatment with L-theanine reduced the serum activity of CK (Fig. 1I) and LDH (Fig. 1J) in I/R mice and that these effects of L-theanine were partially abolished by AG490 (Fig. 1I, F_3,16_ = 61.42, *p* < 0.01; Fig. 1J, F_3,16_ = 61.95, *p* < 0.01). In conclusion, L-theanine ameliorated I/R-induced myocardial injury and protected cardiac function, which was abolished by inhibition of JAK2. This suggests that the therapeutic effect of L-theanine may be related to activation of the JAK2/STAT3 pathway.

### Administration of L-theanine alleviated I/R-induced cellular apoptosis in ischemic cardiac tissue

**Fig. 2:**
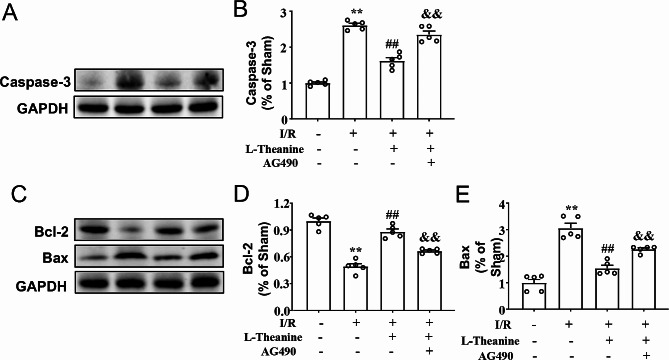
Effect of L-theanine on cardiac apoptosis induced by I/R. **(A, B)** Representative images and quantitative analysis showed that pretreatment with L-theanine (10 mg/kg) prevented the I/R-induced increase in the expression levels of caspase-3 in ischemic cardiac tissue (*n* = 5, **p < 0.01 vs. sham treatment, ##*p* < 0.01 vs. I/R, &&*p* < 0.01 vs. I/R + L-theanine) **(C-E)** Representative images and quantitative analysis showed that pretreatment with L-theanine (10 mg/kg) reduced the I/R-induced decrease in the expression levels of Bcl-2 **(C, D)** as well as the I/R-induced increase in the expression levels of Bax **(D, E)** in ischemic heart tissue (*n* = 5, **p < 0.01 vs. sham treatment, ##*p* < 0.01 vs. I/R, &&*p* < 0.01 vs. I/R + L-theanine). Data are expressed as mean ± SEM.

**Fig. 3:**
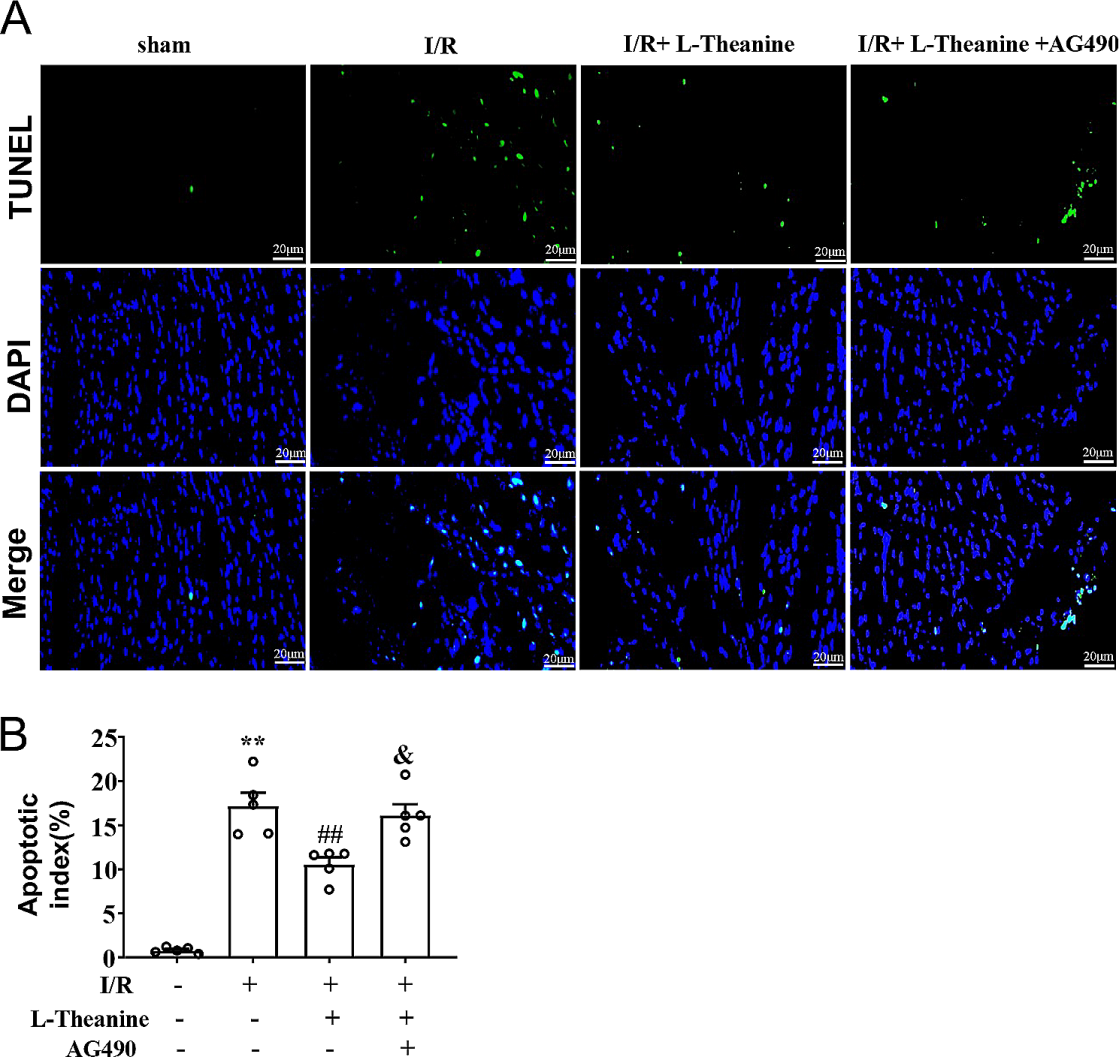
**(A, B)** Representative images **(A)** and quantitative analyses **(B)** showed that pretreatment with L-theanine (10 mg/kg) prevented the I/R-induced increase in the number of TUNEL-positive cells in ischemic heart tissue (*n* = 5, **p < 0.01 vs. sham treatment, ##*p* < 0.01 vs. I/R, &*p* < 0.05 vs. I/R + L-theanine). Scale bar: 20 μm. Data are expressed as mean ± SEM.

To investigate the effect of L-theanine on I/R-induced cellular apoptosis in ischemic heart tissue of mice, we next examined the changes in the expression of anti-apoptotic protein Bcl-2 and pro-apoptotic protein Bax and caspase-3 in the ischemic heart tissue of mice treated with L-theanine or I/R (Fig. 2A, D). Western blot results showed that pretreatment with L-theanine (10 mg/kg) resulted in a remarkable decrease in the expression of the pro-apoptotic protein caspase-3 (Fig. 2B) and Bax (Fig. 2E) compared with the I/R group, and a remarkable increase in the expression of the anti-apoptotic protein Bcl-2 (Fig. 2C) in the ischemic heart tissue of the I/R-treated mice, whereas these effects of L-theanine were partially abolished by AG490 (Fig. 2B, F_3,16_ = 100.50, *p* < 0.01; Fig. 2C, F_3,16_ = 64.05, *p* < 0.01; Fig. 2E, F_3,16_ = 46.58, *p* < 0.01). TUNEL analysis showed that pretreatment with L-theanine reduced the percentage of apoptotic cells in the ischemic heart tissue of I/R mice, which was abolished by the application of AG490 (Fig. 3A, B, F_3,16_ = 48.89, *p* < 0.05). These results demonstrate that pretreatment with L-theanine attenuated I/R-induced cellular apoptosis in the ischemic heart of mice through activation of the JAK2/STAT3 pathway.

### L-theanine reduces I/R-induced oxidative stress in mouse cardiac tissue

**Fig. 4:**
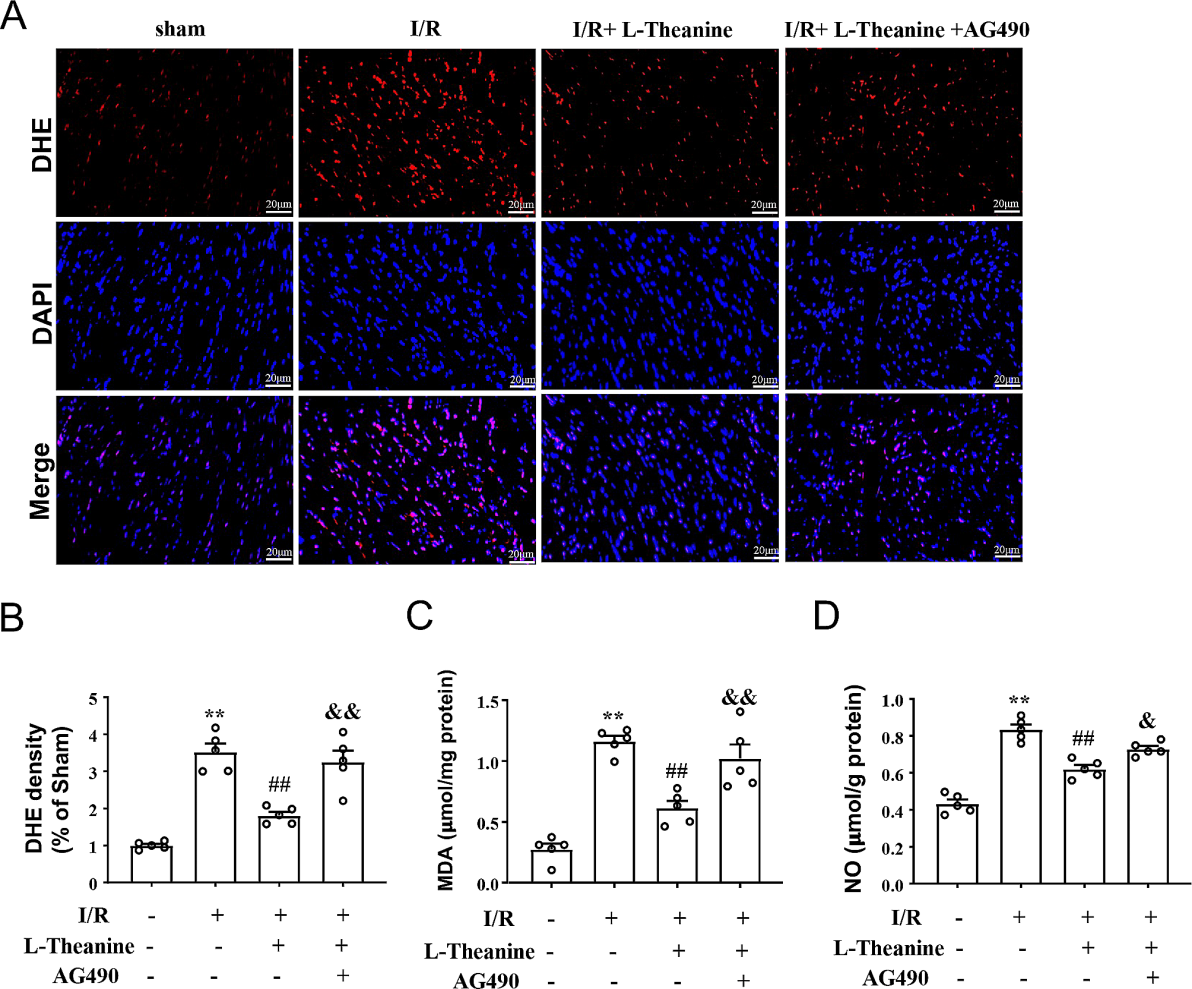
Effect of L-theanine on pro-oxidant stress parameters in myocardial tissue of I/R mice. **(A, B)** Representative images **(A)** and quantitative analysis **(B)** showed that L-theanine pretreatment (10 mg/kg) prevented the I/R-induced increase in the number of DHE-positive cells in ischemic cardiac tissue (*n* = 5, **p < 0.01 vs. sham treatment, ##*p* < 0.01 vs. I/R, &&*p* < 0.01 vs. I/R + L-theanine). **(C, D)** Quantitative analysis showed that L-theanine pretreatment prevented the I/R-induced increase in MDA **(C)** and NO **(D)** levels in ischemic heart tissue (*n* = 5, **p < 0.01 vs. sham treatment, ##*p* < 0.01 vs. I/R, &*p* < 0.05 or &&*p* < 0.01 vs. I/R + L-theanine). Scale bars: 20 μm. Data are expressed as mean ± SEM.

**Fig. 5:**
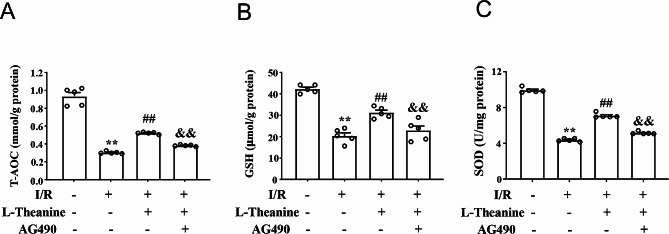
Effect of L-theanine on antioxidant stress parameters in myocardial tissue of I/R mice. **(A-C)** Quantitative analysis showed that L-theanine pretreatment (10 mg/kg) prevented the I/R-induced decrease in T-AOC **(A)**, GSH **(B)**, and SOD **(C)** levels in ischemic cardiac tissue (*n* = 5, **p < 0.01 vs. sham treatment, ##*p* < 0.01 vs. I/R, &&*p* < 0.01 vs. I/R + L-theanine). Data are expressed as mean ± SEM.

To investigate the role of L-theanine in I/R-induced oxidative stress in ischemic hearts, we first stained the heart tissue of mice treated with L-theanine or I/R with fluorescent DHE markers. The results showed that pretreatment with L-theanine (10 mg/kg) significantly reduced the I/R-induced increase in the number of DHE-positive cells in the ischemic heart tissue of I/R mice, and this effect of L-theanine was abolished by AG490 (Fig. 4A, B, F_3,16_ = 35.87, *p* < 0.01). Next, we examined the effect of L-theanine on oxidative stress markers GSH, T-AOC, MDA, NO, and SOD in ischemic heart tissue of mice. Our results showed that pretreatment with L-theanine significantly decreased the I/R-induced increase in MDA (Fig. 4C) and NO (Fig. 4D) levels in the ischemic heart tissue of mice compared with the I/R group, which was partially abolished by treatment with AG490 (Fig. 4C, F_3,16_ = 30.57, *p* < 0.01; Fig. 4D, F_3,16_= 58.52, *p* < 0.05). The antioxidant enzymes GSH, T-AOC, and SOD are known to negatively regulate oxidative stress and reduce severe oxidative damage to cells. Our data showed that pretreatment with L-theanine significantly prevented the I/R-induced decrease in the levels of T-AOC (Fig. 5A), GSH (Fig. 5B), and SOD (Fig. 5C) in the ischemic myocardial tissue of mice, which were also abolished by treatment with AG490 (Fig. 5A, F_3,16_ = 162.2, *p* < 0.01; Fig. 5B, F_3,16_ = 45.74, *p* < 0.01; Fig. 5C, F_3,16_ = 609.10, *p* < 0.01). These results demonstrate that pretreatment with L-theanine attenuates I/R-induced oxidative stress in the ischemic heart of mice by regulating the JAK2/STAT3 signaling pathway.

### L-theanine activates the JAK2/STAT3 signaling pathway in vivo

**Fig. 6:**
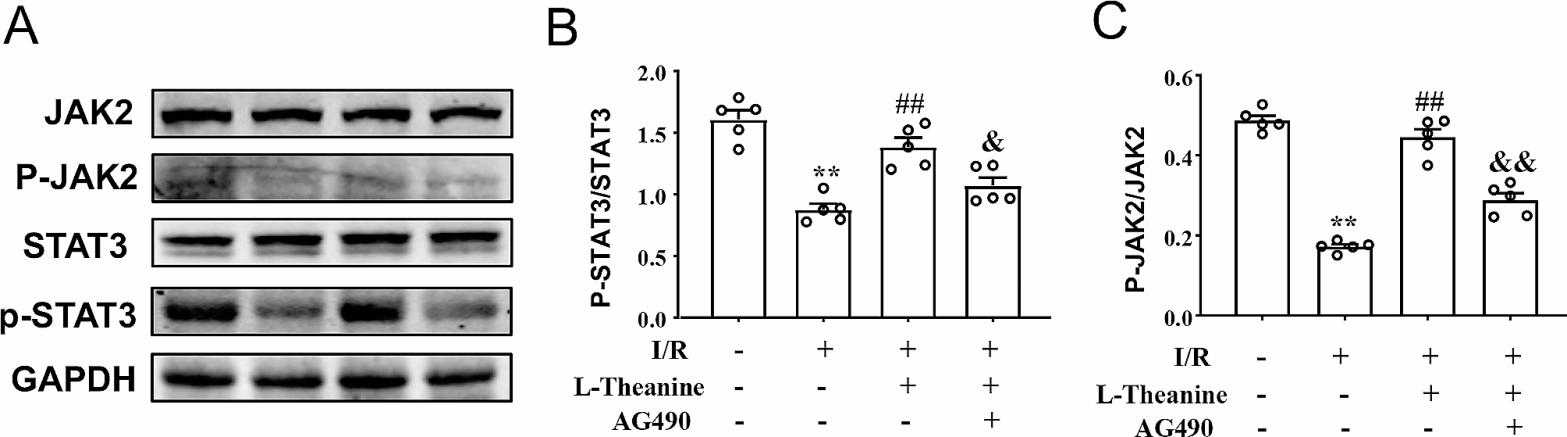
L-theanine activated the JAK2/STAT3 signaling pathway in myocardial tissue from I/R mice. **(A)** Protein expression was measured by immunoblotting assay. The results showed that L-theanine pretreatment activated the expression levels of p-JAK2 and p-STAT3 in ischemia heart tissue. **(B, C)** Quantitative analysis showed that L-theanine pretreatment (10 mg/kg) markedly increased the p-JAK2/JAK2 ratio **(C)** and the p-STAT3/STAT3 ratio **(B)** in I/R-induced ischemic cardiac tissues (*n* = 8, **p < 0.01 vs. sham treatment, ##*p* < 0.01 vs. I/R, &*p* < 0.05 or &&*p* < 0.01 vs. I/R + L-theanine). Data are expressed as mean ± SEM.

To investigate the mechanisms of the cardioprotective effect of L-theanine at MIRI, we examined the effect of L-theanine on JAK2/STAT3 signaling pathways in ischemic heart tissue, which have been shown to be involved in IR-induced oxidative stress and apoptosis (Fig. 6A). We found that pretreatment with L-theanine prevented the IR-induced decrease in phosphorylation levels of JAK2 (Fig. 6A, B) and STAT3 (Fig. 6A, C) in ischemic heart tissue of mice, and these effects of L-theanine were abolished by the application of AG490 (Fig. 6B, F_3,16_ = 24.01, *p* < 0.05; Fig. 6C, F_3,16_ = 91.89, *p* < 0.01). These results suggest that L-theanine protects the ischemic heart of mice from IR-induced damage, at least in part through activation of the JAK2/STAT3 pathway.

## Discussion

I/R injury is the most important process in ischemic heart disease such as myocardial infarction (Hausenloy and Yellon 2013). Although significant progress has been made in understanding ischemic heart disease in recent decades, there is still no effective therapeutic approach to prevent MIRI (Yellon and Hausenloy 2007). In the present study, we investigated the pharmacological effects and mechanisms of L-theanine administration on MIRI in mice a myocardial ischemia-reperfusion model. Our data showed that pretreatment with L-theanine significantly reduced myocardial infarct size, inflammation, oxidative stress, and apoptosis in I/R mice and that the JAK2/STAT3 signaling pathway was closely associated with the cardioprotective effects of L-theanine. These results suggest that L-theanine could be developed as a candidate for the treatment of I/R injury.

Oxidative damage caused by the accumulation of reactive oxygen species (ROS) is recognized as a crucial pathophysiological mechanism in MIRI (Shilo et al. 2021; Li et al. 2022a, b). In cells deprived of oxygen, a limited amount of ROS is produced in the mitochondria and eliminated by the intracellular antioxidant system (Hare 2001). After reperfusion, myocardial ischemia results in high production of ROS in the cell, which overwhelms the defense system and leads to oxidative stress or cell damage (Ferrari et al. 1990). ROS-induced oxidative stress is manifested by an increase in the lipid peroxidation product MDA. As a degradation product, MDA has been used as a biomarker for lipid peroxidation for several decades (Nielsen et al. 1997). Fortunately, the antioxidant GSH, a tripeptide common in plants and animals, is a vital compound of the antioxidant system and can mitigate the side effects of ROS (Galant et al. 2011). In addition, SOD is one of the most important antioxidant enzymes that catalyzes the dismutation of superoxide into oxygen and hydrogen peroxide, and is considered a frontline against potential free radicals that cause oxidative stress (White et al. 1994). Recent studies have shown that L-theanine attenuates hepatic ischemia-reperfusion injury by decreasing ROS and MDA levels and increasing levels of the antioxidant GSH (Küçükaslan et al. 2021). In addition, pretreatment with L-theanine has been reported to attenuate ethanol-induced damage to hepatocytes by restoring GSH levels and increasing the activity of SOD, thereby improving the antioxidant capacity of hepatocytes (Li et al. 2012). L-theanine has also been reported to increase the activities of SOD and catalase by up-regulating SOD mRNA expression in rat heart (Xuefeng et al. 2017). In our study, the production of ROS and MDA increased in cardiac tissues that had suffered I/R injury, while the levels of GSH and SOD decreased. These changes were prevented by pretreatment with L-theanine. It has been reported that L-theanine can modulate the JAK/STAT3 signaling pathway to attenuate angiotensin II-induced proliferation and migration of vascular smooth muscle cells (Ben et al. 2018). Moreover, activation of the JAK2/STAT3 pathway has been shown to be reduced in ischemic heart tissue after MIRI (Wang et al. 2016). There is increasing evidence that JAK2/STAT3 pathway activation can attenuate I/R injury (Das et al. 2012; Yang et al. 2013). Our data showed that L-theanine attenuated the magnitude of oxidative stress in MIRI mice and increased the antioxidant capacity of cardiomyocytes in part via the JAK2/STAT3 signaling pathway.

It is known that the stimulus of ischemia-reperfusion can lead to the production of free radicals in ischemic cardiac tissue (Chen et al. 1991), which trigger oxidative stress by promoting lipid peroxidation and membrane damage through the cross-linking of proteins, lipids, and nucleic acids (Jiwajinda et al. 2002). NO is a small free radical molecule produced by inducible nitric oxide synthase (iNOS) in various cells and tissues (Zhao et al. 1996). NO from L-arginine in cardiomyocytes has been identified as an endogenous regulator of myocardial function (Massion et al. 2003), and pathological overproduction of NO promotes the development of inflammation (Hofseth 2008). Recent studies have shown that L-theanine reduces the overproduction of NO (Di et al. 2010). It has been shown that L-theanine treatment significantly inhibited inflammatory responses and attenuated intestinal barrier dysfunction in a sodium dextrose sulfate-induced colitis model in C57BL/6J mice by decreasing the transcript levels of iNOS in the colon (Wang et al. 2020). Moreover, it was reported that L-theanine treatment could alleviate osteoarthritis (OA)-induced inflammation and injury by reducing the levels of inducible nitric oxide synthase (iNOS) and NO in an IL-1β-induced OA rat model in vivo and in vitro (Bai et al. 2020). Our data showed that NO levels and oxidative stress in ischemic myocardial tissue were significantly higher in the I/R group, accompanied by a decrease in phosphorylated JAK2 and STAT3. However, pretreatment with L-theanine decreased NO levels and significantly activated the JAK2/STAT3 signaling pathway in ischemic tissue, and application of the JAK2 inhibitor AG490 abolished the antioxidant stress effect of L-theanine. The JAK2-mediated cardioprotective effect of L-theanine in I/R mice is consistent with previous studies showing that activation of the JAK2/STAT3 signaling pathway attenuates I/R injury in the heart (Yang et al. 2013).

It has long been assumed that the loss of cardiac cells in response to ischemia/reperfusion injury is due to necrotic cell death. However, studies have shown that apoptosis is an essential component of cell loss during reperfusion after myocardial infarction (Haunstetter and Izumo 1998; Olivetti et al. 1996; Saraste et al. 1997; Isik et al. 2021; Işık and Fırat 2021). Previous studies have shown that cardiac myocytes undergo apoptosis in response to various stimuli such as hypoxia (Gottlieb et al. 1994), reperfusion, and oxidative stress (Fliss and Gattinger 1996). Apoptosis, which is mediated by pro-apoptotic Bax and anti-apoptotic Bcl-2 proteins, is part of the mitochondrial apoptosis pathway (Biala and Kirshenbaum 2014). Bax and Bcl-2 have been shown to be involved in oxidative stress and apoptosis in ischemia-reperfusion injury (Maulik et al. 1999; Park and Hockenbery 1996). Recruitment and oligomerization of Bax proteins at the outer mitochondrial membrane (OMM) promotes permeabilization of the OMM, which ultimately leads to loss of mitochondrial Δψm and thus promotes cell death (Konstantinidis et al. 2012). However, Bcl-2 suppressed death by sequestering Bax proteins, preventing permeabilization of the OMM (Jacobson et al. 1993). It has been shown that isolated hearts from Bax knockout mice had a 50% reduction in infarct size (Hochhauser et al. 2003), and transgenic mice overexpressing Bcl-2 in the heart had less I/R injury and fewer apoptotic cells (Chen et al. 2001). In addition, inhibition of caspase-3 was shown to decrease apoptosis in cardiomyocytes and improve cardiac function (Adams et al. 1998). Our results showed that caspase-3 and Bax protein levels were increased and Bcl-2 protein levels were decreased in the ischemic cardiac tissue of the I/R group, accompanied by a decrease in phosphorylated JAK2 and STAT3. However, pretreatment with L-theanine significantly decreased the expression of Bax and caspase-3, increased the expression of Bcl-2, and activated the JAK2/STAT3 signaling pathway, which contributed to the attenuation of apoptosis in ischemic cardiac tissue. Of note, administration of AG490 abrogated the anti-apoptotic effect of L-theanine. This is consistent with previous reports that pretreatment with L-theanine protected H9C2 cells from H2O2-induced apoptosis (Li et al. 2018). Thus, in this study, our research provides strong evidence for the anti-apoptotic effect of L-theanine in I/R mice in vivo. We hypothesize that L-theanine exerts its anti-apoptotic effect in vivo in I/R mice in part through the JAK2/STAT3 pathway, which should be confirmed by further studies.

## Conclusion

The present study has shown that pretreatment with L-theanine enhances JAK2/STAT3-dependent pro-survival signaling and antioxidant responses, thereby effectively attenuating I/R-induced oxidative stress and cardiomyocyte apoptosis. These results provide further insight into the protective role of L-theanine in ischemic heart disease. A limitation of the present study is that we did not investigate the specific targets and molecular mechanisms for the cardioprotective effects of L-theanine in cardiac I/R models under in vitro conditions. Another limitation is that we did not investigate whether the effect of L-theanine depends solely on JAK2/STAT3 signaling in the current study, and further studies are needed to clarify this tissue.

### Electronic supplementary material

Below is the link to the electronic supplementary material.

Supplementary Material 1

Supplementary Material 2

## Data Availability

The original contributions presented in the study are included in the article, further inquiries can be directed to the corresponding author.
