# Supplementary material for: L-theanine alleviates myocardial ischemia/reperfusion injury by suppressing oxidative stress and apoptosis through activation of the JAK2/STAT3 pathway in mice
Source: Mol Med. 2024 Jun 28;30:98. doi: 10.1186/s10020-024-00865-0 (PMC11214244; doi:10.1186/s10020-024-00865-0)
Supplement: Supplementary file 2 — Supplementary Material 2 [file 10020_2024_865_MOESM2_ESM.doc]

Dear editors,

Good day!

We would like to submit the manuscript entitled “**L-theanine alleviates myocardial ischemia/reperfusion injury by suppressing oxidative stress and apoptosis through activation of the JAK2/STAT3 pathway in mice**” to Molecular Medicine. All co-authors have seen and agreed with the contents of this manuscript, and this manuscript is in accordance with the Authorship statement of ethical standards for manuscripts submitted to Molecular Medicine. This work is supported by the Natural Science Foundation of China (81974216 and 81974015), the Science and Technology Project of Nantong City (MS12020016), the Jiangsu Health Commission (Z2021005), and the High Technology Research Project of the Suzhou Science & Technology Division (SKY2021003).

Ischemic heart disease has become a predictive risk factor for adverse cardiovascular events. Despite recent advances, there is no effective therapy to prevent myocardial ischemia-reperfusion injury (MIRI). L-theanine is a unique non-protein amino acid in tea that has multiple actions, including against cardiovascular disease, but little is known about its effects or mechanisms at MIRI. Our results showed that L-theanine pretreatment prevented the MIRI-induced myocardial injury, cardiac apoptosis and oxidative stress in MIRI mice. Of note, L-theanine activated the expression of phosphorylated JAK2 and STAT3 in MIRI-induced cardiac tissue, suggesting that the JAK2/STAT3 pathway plays an essential role in mediating the anti-I/R effects. AG490 (a JAK2 inhibitor) blocked l-theanine-mediated cardioprotection by inhibiting JAK2/STAT3 signaling. Taken together, our results showed that administration of L-theanine exerted its anti-apoptotic and antioxidant effects in part through the JAK2/STAT3 signaling pathway to alleviate MIRI. **We believe that our findings would induce great interest for readers, as L-theanine was found to exhibit cardiac anti-oxidative and anti-apoptotic protection through JAK2/STAT3 pathway in mice model of MIRI, which deepened the understanding of cardio-protective mechanism of l-theanine. This finding may be beneficial to develop drugs for preventing MIRI after percutaneous coronary intervention or coronary artery bypass graft surgery.**

We deeply appreciate your consideration of our manuscript, and are looking forward to receive comments from you and the reviewers. If you have any queries, please don’t hesitate to contact me at this E-mail address: yqscy@126.com.

Sincerely,

Qingsheng You
