# Supplementary material for: L-theanine alleviates myocardial ischemia/reperfusion injury by suppressing oxidative stress and apoptosis through activation of the JAK2/STAT3 pathway in mice
Source: Mol Med. 2024 Jun 28;30:98. doi: 10.1186/s10020-024-00865-0 (PMC11214244; doi:10.1186/s10020-024-00865-0)
Supplement: Supplementary file 1 — Supplementary Material 1 [file 10020_2024_865_MOESM1_ESM.docx]

Dear editors and reviewers

Re: Manuscript MOME-D-23-01232 and Title: L-theanine alleviates myocardial ischemia/reperfusion injury by suppressing oxidative stress and apoptosis through activation of the JAK2/STAT3 pathway in mice

Thank you for your letter and reviewer comments on our manuscript entitled “L-theanine alleviates myocardial ischemia/reperfusion injury by suppressing oxidative stress and apoptosis through activation of the JAK2/STAT3 pathway in mice”. These comments are valuable and very helpful. We have carefully reviewed the comments and made corrections. Following the instructions in your letter, we have uploaded the file of the revised manuscript.

We would like to thank you for allowing us to resubmit a revised version of the manuscript, and greatly appreciate your time and attention.

Sincerely,

Qi Li

Reviewer #1:

1- please define aim

**Response**: Thank you for your comment. We have mentioned the aim of our study in the abstract: L-theanine could be developed as a potential drug to alleviate cardiac damage under MIRI conditions. We investigated its cardioprotective effects and mechanisms in myocardial ischemia-reperfusion injury (MIRI).

2-("Letter to the editor concerning "Most cited 100 articles from Turkey on abdominal wall hernias: a bibliometric study"." Turkish Journal of Surgery 37.2 (2021): 193.) and ("Incomplete or inappropriate endoscopic and radiologic interventions as leading causes of cholangitis." Polish Journal of Surgery 93.6 (2021): 47-52.) suggested studies for the references

**Response**: Thank you for your suggestions, we have cited the references to both articles (PMID: 36169535 and 37275187).

Reviewer #2:

1. How many mice were used for calculation of the results? Are the data presented an average of all mice used or a representative display?

**Response**: We are very grateful to the reviewer for drawing our attention to this problem. As shown in the notes to the figure, our data are the result of a random count of 5 groups of mice and are not representative.

2. Did any animals die in the various groups and excluded from analysis?

**Response**: Thank you for your comment. The model used in the study is the mouse myocardial ischemia-reperfusion injury model. According to the existing studies, this model has a certain mortality rate during the construction process (PMID: 29987819). To ensure the reliability and consistency of the experimental results, we keep the same number of surviving mice in each group.

3. Is there a theory on how exactly does L-theanine exerts its effect on the JAK2/STAT3 pathway? Is it inhibition of activity or effect on transcription? does the antagonist affects phosphorylation?

**Response**: We are very grateful to the reviewer for bringing this issue to our attention. Previous studies have shown that L-theanine regulates the JAK/STAT3 signaling pathway to decrease proliferation and migration of vascular smooth muscle cells induced by angiotensin II. (PMID: 30381668) Antagonists of L-theanine have been shown not to affect JAK2/STAT3 phosphorylation levels. (PMID: 32673665)

4. Last word in conclusion should be issue.

**Response**: We are very grateful to the reviewer for drawing our attention to this problem. We have rewritten the sentence.

6. Is there data on the pharmacodynamics of L-theanine? Can it be administered orally, IV?

**Response**: We are very grateful to the reviewer who asked this question about the L-theanine injection method. Studies have been conducted to detail the pharmacodynamic distribution of L-theanine in the heart and liver of mice. (PMID: 15279898, 25680506 and 22019691) There is strong evidence that L-theanine can enter and exert its effects in the body of mice by intraperitoneal, oral, and intravenous injection. (PMID: 26828717, 32720164 and 23097345).

7. How was the echo done technically ? were the animals sedated?

**Response**: We are very grateful for this question. We performed the cardiac ultrasound examination in mice as follows: Experimental mice were placed in transparent containers filled with isoflurane and cardiac function was assessed using a small animal ultrasound system (VEVO 2100, VisualSonics, Toronto, Canada) after the mice were anesthetized. M-mode recordings were recorded in the short-axis view of the left ventricle at the level of the papillary muscle. Left ventricular ejection fraction (EF) and fractional shortening (FS) were calculated using the following formula: EF= (EDV – ESV)/EDV ×100%; FS= (LVIDd – LVIDs)/LVIDd ×100%, respectively. (PMID: 35734410)

Reviewer #3:

Major comments:

1. It is important to examine whether post-treatment or co-treatment of L-theanine can rescue I/R induced cardiac injuries, as ischemic heart diseases often onset without any symptoms.

**Response**: We greatly appreciate the reviewer’s suggestion. We will further investigate whether post-treatment or co-treatment with L-theanine rescues I/R-induced cardiac injury and further explain the pharmacologic mechanism in future studies.

2. To conclude that the protective effect of L-theanine is mediated by JAK2-STAT3 pathway, it is necessary to examine the role of STAT3 pathway in the current model. Does STAT3 inhibitor treatment affect the protective role of L-theanine in the current model?

**Response**: We are very grateful to the reviewer for bringing this matter to our attention. It has been reported that STAT3 inhibitors alone have no significant therapeutic efficacy compared to other groups (PMID: 32673665). One possible explanation is the following: STAT3 inhibitors have an appreciable inhibitory effect only when the expression of STAT3 protein is exceptionally high, whereas in this study, the expression of P-STAT3 was extremely low in the hearts of mice after myocardial ischemia/reperfusion. Consequently, the effect of a STAT3 inhibitor might not be significant. However, after L-theanine administration, P-STAT3 was activated and its expression increased significantly, leading to a favorable therapeutic effect of STAT3 inhibitors. Of course, this could be considered a limitation of our study, and we intend to further investigate the therapeutic effect of STAT3 inhibitors alone in future studies.

Minor comments:

1. The downstream pathways of JAK2 include STAT3 and PI3K. Does the current I/R model regulate IRS-1-PI3K pathway (including phosphorylation of AKT and ERK). If so, does L-theanine treatment regulate those molecules in I/R?

**Response**: We are very grateful to the reviewer for drawing our attention to this problem. First, based on the existing studies, there is currently no report indicating an effect of L-theanine on the PI3K signaling pathway in the cardiac tissue of mice with ischemia/reperfusion model. In addition, whether the I/R model regulates the IRS-1-PI3K signaling pathway (including phosphorylation of AKT and ERK) remains to be experimentally demonstrated. This study focuses exclusively on how L-theanine alleviates myocardial ischemia/reperfusion injury through its effect on the JAK2/STAT3 signaling pathway. Even if L-theanine were to affect the IR-induced PI3K signaling pathway, our study used a tightly controlled method with four groups: Sham treatment, IR, L-theanine + IR, and L-theanine+IR+AG490. Nevertheless, we demonstrated that L-theanine was effective against myocardial ischemia-reperfusion injury in mice by affecting the JAK2/STAT3 signaling pathway. In conclusion, we would like to thank the reviewer for his suggestion, which gave us an excellent idea for future in-depth research on L-theanine, and we will incorporate these considerations into our future studies and improve our experimental design.

2. In figure 2A, does casapase-3 mean total or cleaved form? Please include both total and cleaved cas-3 blots.

**Response**: We are very grateful to the reviewer for bringing this issue to our attention. In Figure 2A, the casapase-3 form cleaved. The discussion on this issue is presented below: Caspases are a family of protein hydrolases that are closely related to apoptosis and are found as caspase precursor zymogens in most animal cells. Cleaved caspase-3 is the activated form of caspase-3, which is only found in cells undergoing apoptosis. Moreover, cleaved caspase-3 is not present in cells that are not undergoing apoptosis or have already undergone necrosis. This has led to numerous studies showing that cleaved caspase-3 is a more accurate and specific marker for apoptotic cells. (PMID: 32246616, 29101802 and 21274568) In future studies, we will discuss in detail how L-theanine affects the expression of cleaved and total caspase-3 under conditions of myocardial ischemia-reperfusion injury in mice.

3. Please include the product information of antibodies used in this study in the method section.

**Response**: Thank you for your comment. As suggested by the reviewer, we have added more details to the product information of the antibodies used in this study in the Method section under "Western blot".

Reviewer #4:

Major comments:

1) It would be helpful to know whether the beneficial effects of L-theanine persist beyond 24h. I recommend the authors perform a limited subset of their experiments (e.g., EF and ischemic area over at risk area) at a later time point (~7d).

**Response**: We greatly appreciate your suggestions. In our future experiments, we plan to thoroughly investigate differences in cardiac function, infarct area, and other key indicators of the mouse heart during ischemia and at different time points of reperfusion, including one day, seven days, and beyond. These additional studies will provide valuable insights into the specific mechanisms of L-theanine-mediated ischemia/reperfusion injury in the heart.

2) While the authors' results are very intriguing, it would be very helpful to know if L-Theanine administration at time of or immediately after ischemia improves outcomes. This would significantly increase the translational relevance of their work.

**Response**: We are very grateful for this suggestion. Having found that pretreatment with L-theanine has a cardioprotective effect in IR-induced mice prior to ischemia, we have developed a preliminary experimental protocol to test whether administration of L-theanine at the time of or immediately after ischemia improves cardiac function and limits infarct size in mice. This research will serve as the basis for future studies aimed at elucidating the pharmacological mechanisms of L-theanine in the treatment of myocardial ischemia/reperfusion injury and ultimately support the clinical development of drugs for this condition.

3) The authors should note whether the histology, echocardiography, and molecular biology (Westerns) were performed in a blinded fashion.

**Response**: We thank the reviewers for their great attention to this important topic. To ensure scientific accuracy, all experimental data were carefully collected in a blinded fashion and each result was verified by numerous tests.

4) In Figure 3, the authors should explain their methods for the "Apoptotic index" utilized for quantification.

**Response**: We are very grateful to the reviewers for their valuable feedback. The apoptosis index was determined by carefully viewing TUNEL-stained heart tissue sections under the microscope and calculating the percentage of positive cells (indicated by green particles in the nucleus) compared to the total number of cells (indicated by blue particles in the nucleus) within the field of view. (PMID: 22307460)

5) The authors should include full Western Blot gels as supplemental figures.

**Response**: We greatly appreciate the reviewer’s feedback. All of our Western blot gels are intercepted in full Western Blot gels and we are always willing to upload them upon request.

Minor comments

1) The authors should include page numbers and line numbers to assist with review.

**Response**: We appreciate your suggestion and have provided the full text with page and line numbers to facilitate reviewers.

2) On page 20, lines ~31-39, the authors seem to have misstated that L-theanine pre-treatment enhances apoptosis. This should read, "reduces apoptosis."

**Response**: We are very grateful for this suggestion and have re-written the sentence.
